# The Influence of Cold Atmospheric Pressure Plasma-Treated Media on the Cell Viability, Motility, and Induction of Apoptosis in Human Non-Metastatic (MCF7) and Metastatic (MDA-MB-231) Breast Cancer Cell Lines

**DOI:** 10.3390/ijms22083855

**Published:** 2021-04-08

**Authors:** Dominik Terefinko, Anna Dzimitrowicz, Aleksandra Bielawska-Pohl, Aleksandra Klimczak, Pawel Pohl, Piotr Jamroz

**Affiliations:** 1 Department of Analytical Chemistry and Chemical Metallurgy, Faculty of Chemistry, Wroclaw University of Science and Technology, Wybrzeze St. Wyspianskiego 27, 50-370 Wroclaw, Poland; pawel.pohl@pwr.edu.pl (P.P.); piotr.jamroz@pwr.edu.pl (P.J.); 2 Laboratory of Biology of Stem and Neoplastic Cells, Hirszfeld Institute of Immunology and Experimental Therapy, Polish Academy of Sciences, Weigla 12, 53-114 Wroclaw, Poland; aleksandra.bielawska-pohl@hirszfeld.pl (A.B.-P.); aleksandra.klimczak@hirszfeld.pl (A.K.)

**Keywords:** non-thermal plasma, reactive oxygen and nitrogen species, biological activity, breast cancer

## Abstract

Breast cancer remains the most common type of cancer, occurring in middle-aged women, and often leads to patients’ death. In this work, we applied a cold atmospheric pressure plasma (CAPP)-based reaction-discharge system, one that is unique in its class, for the production of CAPP-activated media (DMEM and Opti-MEM); it is intended for further uses in breast cancer treatment. To reach this aim, different volumes of DMEM or Opti-MEM were treated by CAPP. Prepared media were exposed to the CAPP treatment at seven different time intervals and examined in respect of their impact on cell viability and motility, and the induction of the apoptosis in human non-metastatic (MCF7) and metastatic (MDA-MB-231) breast cancer cell lines. As a control, the influence of CAPP-activated media on the viability and motility, and the type of the cell death of the non-cancerous human normal MCF10A cell line, was estimated. Additionally, qualitative and quantitative analyses of the reactive oxygen and nitrogen species (RONS), generated during the CAPP operation in contact with analyzed media, were performed. Based on the conducted research, it was found that 180 s (media activation time by CAPP) should be considered as the minimal toxic dose, which significantly decreases the cell viability and the migration of MDA-MB-231 cells, and also disturbs life processes of MCF7 cells. Finally, CAPP-activated media led to the apoptosis of analyzed cell lines, especially of the metastatic MDA-MB-231 cell line. Therefore, the application of the CAPP system may be potentially applied as a therapeutic strategy for the management of highly metastatic human breast cancer.

## 1. Introduction

The incidence of cancer in the world population is growing. In the group of females, breast cancer is one of the most common types of cancers, following lung cancer [1,2]. According to the National Breast Cancer Foundation, 276,480 new cases of invasive breast cancer are predicted to be diagnosed in 2020 [2]. Additionally, a highly metastatic character of breast cancer decreases the survival rate and the convalescence perspective for diagnosed patients, and is often associated with a lack of effective treatment modalities. Nowadays, several therapies, including modern chemotherapy, radiotherapy, or immunotherapy, are used to treat breast cancer, but they are often associated with pain, infections, amputations, deformations, and other side effects [3]. For that reason, there is a high need for new, fast, and non-invasive therapies used for breast cancer treatment.

An encouraging and quite new approach to breast cancer treatment is a targeted drug-based therapy [4]. This therapy can be supported by gold nanoparticles (AuNPs), used as carriers for specific drugs [5,6]. Unique optical and structural properties of AuNPs in addition to their high surface-to-volume ratio enable to attach various inhibitors or proteins to their surface and carry them to a defined target in the body [6]. As was found by Devi et al. [6], a target-based therapy is characterized by very promising rates of survival and low side effects after its application [6]. Another method used for reducing breast cancer volume is hyperthermia [6]. In this process, the temperature of tissues is locally raised by an artificial heat source, leading to damage of cancerous cells [7,8]. A very promising strategy for making the hyperthermia process more effective is the application of magnetic nanoparticles [7,8]. In magnetic hyperthermia, a magnetic nanofluid is transported via blood to a targeted tissue, and then the local heat is induced in a radiofrequency magnetic field [8]. From a long-term perspective, such application of magnetic nanoparticles inside tissues can, however, be hazardous because of the absorption of nanoparticles into the intracellular environment, especially in the case of lymphatic tissues of the intestine, which lead to the generation of the oxidative stress, cell destruction, and genotoxicity [9]. A remedy to this drawback could be the utilization of cold atmospheric pressure plasmas (CAPPs), those being a new, very promising and effective treatment modality in breast cancer therapy [10,11,12,13,14,15,16,17,18,19,20,21,22].

Significant biological effects of CAPP were identified at the end of the last decade as a result of reactive oxygen and nitrogen species (RONS) production. Because hydrogen peroxide (H_2_O_2_), nitric oxide (NO), nitrate (NO_3_^−^), nitrite (NO_2_^−^), ammonia (NH_4_^+^) ions, hydroxyl (OH^•^), and hydrogen (H^•^) radicals are produced during the CAPP operation with water or cell culture media [10,23], the CAPP-based approach is used in a wide range of applications such as wound healing [24], inactivation of parasites and foreign organisms [25], dentistry [26], seed germination [27], nanoparticles synthesis [28], blood coagulation [29], and so on. Concerning breast cancer therapy, several reaction-discharge systems have been developed and studied, including dielectric barrier discharges (DBDs) [12,13,14,15,17,19,20,21], atmospheric pressure plasma jets (APPJs) [10,16,18], and radio-frequency discharge [22]. CAPP sources applied in these systems have been used for the direct [10,11,13,14,16,17,18,19,20,21,22] or indirect [12,14,15,20] treatment of cell lines. In the first case, selected cell lines, i.e., MDA-MB-231 [10,11,12,14,16,20,22], MCF-7 [10,13,15,17,18,21], SKBR3 [15], AMN3 [21], AMJ13 [19,21], and MCF10A [11,18], were directly treated by proper CAPP sources for specific times. Next, the effect of such direct CAPP treatment on the cell lines’ viability was assessed; however, it exhibited some limitations, mostly related to its insufficient efficiency [10,11,13,14,16,17,18,19,20,21,22]. Additionally, in some cases, the application of the direct CAPP treatment on cell lines was restricted due to problems with the transportation of the whole reaction-discharge system to a place of cell line irradiation. In most cases, the CAPP-based reaction-discharge systems were expensive and not very mobile. 

A remedy to these drawbacks was the indirect CAPP-based method used for water [12] or cell culture media [14,15,20] activation. The resultant CAPP-activated liquids (DMEM, L-15/DMEM, DMEM/F-12, RPMI 1640, serum free medium) were used then in the MDA-MB-231 [10,11,12,14,16,20,22], MCF-7 [10,13,15,17,18,20,21], MCF10A [11,15,18,20], and AMJ13 [19] and SKBR3 [15] cell lines treatment. Indeed, they could be easily stored and transported for further application to cancerous cell lines, causing their death due to an efficient transfer of RONS [14]. In addition, the selectivity of the CAPP-activated media was that the cancerous cell lines were more vulnerable to the oxidative stress caused by RONS than the normal cell lines, which were not so sensitive to this oxidative stress [30]. What is more, in the case of the application of the CAPP-activated media towards the cancerous cell lines, there was no physical effect on the cell lines associated with the damages caused by a gas flux (CAPP discharge gas) and the electromagnetic field (from a CAPP source) [31,32].

In this study, we examined the influence of two CAPP-treated media, including DMEM and Opti-MEM, on the biological activity of three cell lines: The highly metastatic human breast cancer MDA-MB-231 cell line, the non-invasive human breast cancer MCF7 cell line, and the non-cancerous human normal MCF10A cell line. To reach this aim, we developed and used a special portable CAPP-based reaction-discharge system, which had already been successfully applied for the activation of normal human skin cell lines [33]. To the best of our knowledge, this was the first mobile and inexpensive in-operation CAPP-based reaction-discharge system in which a DBD plasma jet was applied for human cell line treatment. A broad spectrum of confirmed utilizations of the studied CAPP-based reaction-discharge system deserves special attention. In the previous study, it was described that the direct CAPP-treatment of human normal cell lines results in the enhancement of their proliferation ability, supporting wound healing processes. From this perspective, we suggest that the modulation of the CAPP-treatment regimen, as well as the treatment time and the targeted liquid environment, may influence the cancer biological response. Furthermore, we have studied the scavenger effect of the fetal bovine serum (FBS) addition to the CAPP-treated media in order to assess its anticancer activity towards the analyzed human cell lines. We experimentally inspected how the quantitative and qualitative composition of the produced reactive oxygen species can provoke a different biological response. Finally, we performed a detailed evaluation of processes and interactions occurring in the CAPP-treated media interfaces to better assess the composition of major active components and correlate them with biological studies.

## 2. Results and Discussion

### 2.1. Effect of the CAPP-Activated Media on the Defined Biological Activities

The CAPP-activated media, including DMEM and Opti-MEM, were used for the assessment of the selected biological functions of the target breast cancer cell lines such as cell viability, cell migration, and cell death type (apoptosis/necrosis).

#### 2.1.1. Effect of the CAPP-Activated Media on Cell Viability

**Figure 1:**
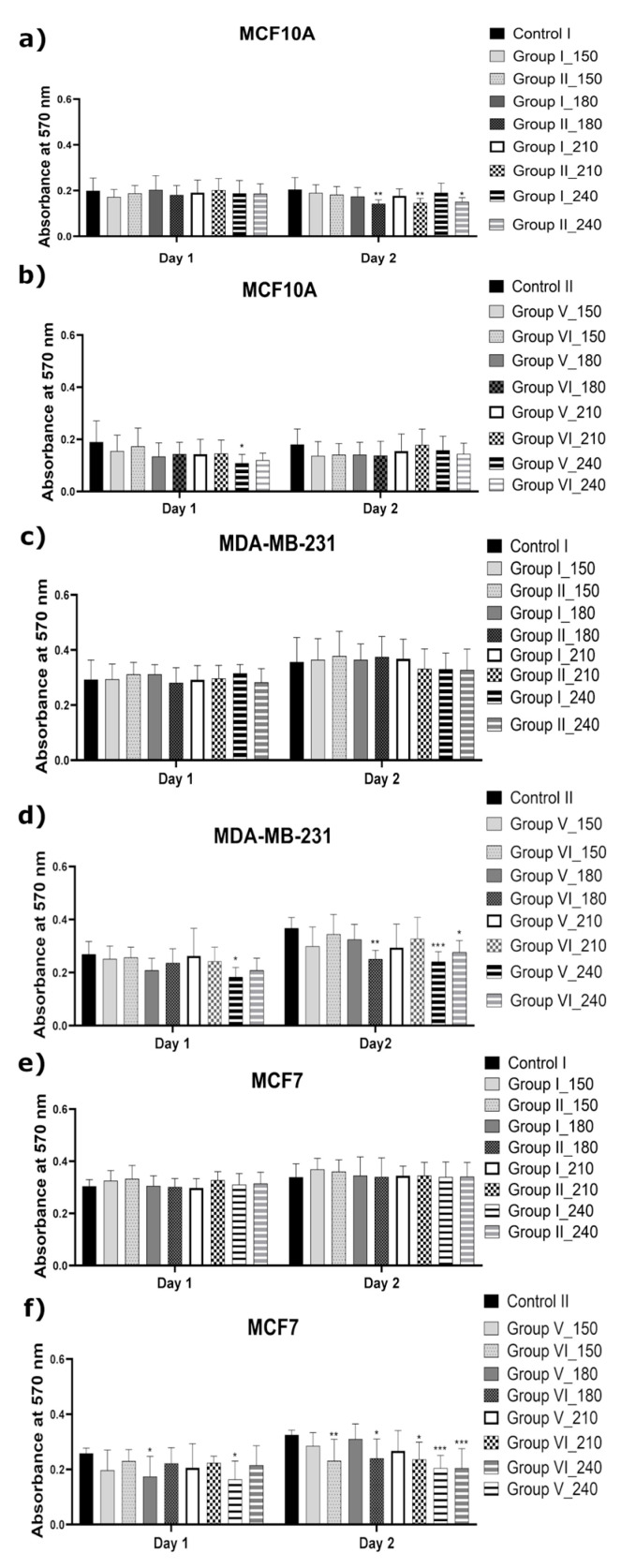
The MTT assay for the proliferation of human metastatic breast cancer (MDA-MB-231), human non-metastatic breast cancer (MCF7), and non-cancerous human normal (MCF10A) cell lines, incubated from 0 to 2 days in the cold atmospheric pressure plasma (CAPP)-activated media (exposure times of 150 s, 180 s, 210 s, or 240 s). (**a**,**c**,**e**) The MCF10A, MDA-MB-231, and MCF7 cell lines incubated in 3.0 mL (Group I) or in 1.5 mL (Group II) of the CAPP-activated DMEM. (**b**,**d**,**f**) The MCF10A, MDA-MB-231, and MCF7 cell lines incubated in 3.0 mL (Group V) or 1.5 mL (Group VI) of the CAPP-activated Opti-MEM. In both cases, fetal bovine serum (FBS) was added to the analyzed medium before the CAPP treatment. As a control, cells untreated by a CAPP-activated medium were used (Control I as the untreated DMEM and Control II as the untreated Opti-MEM). Data are means ± SD values for three independent experiments conducted in triplicates. The statistical calculation was performed by a comparison of all investigated groups versus the control using one-way analysis of variance (ANOVA) with the Dunnett’s post hoc test. (* *p* < 0.01; ** *p* < 0.001; *** *p* < 0.0004).

**Figure 2:**
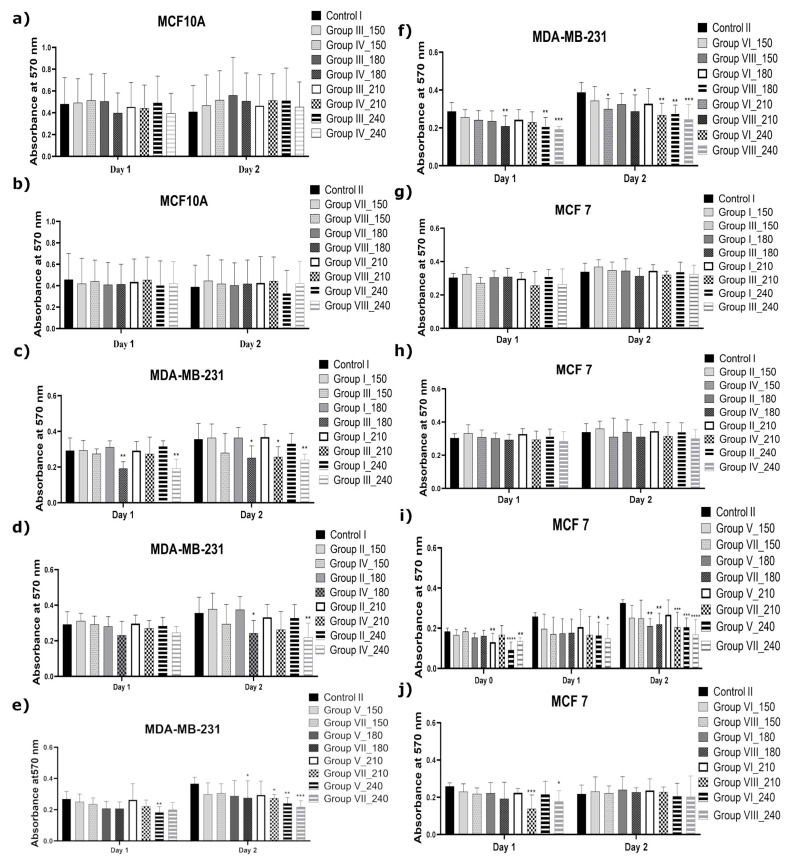
The comparison of the impact of the addition of FBS after the production of the CAPP-activated medium on the proliferative ability of human metastatic breast cancer (MDA-MB-231), human non-metastatic breast cancer (MCF7), and non-cancerous human normal (MCF10A) cell lines. The cell lines were incubated in the CAPP-activated medium obtained by CAPP treatment for 150 s, 180 s, 210 s, or 240 s. (**a**,**c**,**g**) The MCF10A, MDA-MB-231, and MCF7 cell lines treated with 3.0 mL of the CAPP-activated DMEM with the FBS (Group I) or without the FBS (Group III); (**a**,**d**,**h**) the MCF10A, MDA-MB-231, and MCF7 cell lines treated with 1.5 mL of the CAPP-activated DMEM with the FBS (Group II) or without the FBS (Group IV); (**b**,**e**,**i**) the MDA-MB-231 and MCF7 cell lines treated with 3.0 mL of the CAPP-activated Opti-MEM with the FBS (Group V) or without the FBS (Group VII); (**b**,**f**,**j**) the MDA-MB-231 and MCF7 cell lines treated with 1.5 mL of the CAPP-activated Opti-MEM with the FBS (Group VI) or without the FBS (Group VIII). A culture medium that was not treated with CAPP was used as control (Controls I and II). Data are means ± SD values for three independent experiments conducted in triplicates. The statistical calculation was performed by the comparison of all the investigated groups versus the control using one-way ANOVA with the Dunnett’s post hoc test (* *p* < 0.013; ** *p* < 0.0014; *** *p* < 0.0002; **** *p* < 0.0001).

Cell viability after the application of the CAPP-activated medium was assessed using an MTT assay. The minimal toxic dose for the normal cell line (MCF10A) was estimated as the medium treatment time by CAPP, which resulted in decreasing the cell line viability. The disorders in cell viability of the MCF10A cells were noted when the FBS was present in both culture media during the CAPP-activation process (Figure 1a,b). On the other hand, it was found that the MCF10A cells showed high resistance to the CAPP-activated media, where the FBS was introduced after the preparation step (Figure 2a,b). Similar observations were found by other scientists, but in these cases the normal human cell lines (MCF10A) were directly treated by CAPP [15,18,20]. A preliminary screening of the time of the CAPP exposition on the cell culture media showed that the application of these CAPP-activated media (CAPP treatment time within 45–150 s) on the human breast cancer cell lines such as MDA-MB-231 and MCF7 did not affect their mitochondrial activity and proliferation rate (data not shown). Based on these observations, the analyzed medium was exposed to CAPP for 150 s, 180 s, 210 s, or 240 s (Figure 1). The selected CAPP-activated media (groups V and VI) exhibited a significant reduction in the mitochondrial activity of the MDA-MB-231 and MCF7 cell lines (* *p* < 0.01; ** *p* < 0.001; *** *p* < 0.0004, Figure 1). In more detail, the CAPP-activated Opti-MEM significantly affected the cell viability of the MDA-MB-231 cell line incubated for one day, when the longest CAPP-treatment time (240 s—* *p* < 0.01) of the analyzed medium was used. During two days of incubation of the MDA-MB-231 cell line in the CAPP-activated Opti-MEM, it exhibited a great impact on a decrease of the cell viability, especially in the case of the CAPP treatment times of 180 s and 240 s (** *p* < 0.001, *** *p* < 0.0004, respectively). Nevertheless, for the MCF7 cell line, a significant reduction in the cell viability following the one-day incubation was observed for the CAPP treatment times of 180 s and 240 s (* *p* < 0.01, ** *p* < 0.01, respectively). Similar effects were observed following the two-day incubation, leading to a more prominent cell viability reduction for the CAPP treatment times of 150 s, 180 s, 210 s, and 240 s (** *p* < 0.001, * *p* < 0.01, * *p* < 0.01, **** *p* < 0.0004, respectively). Based on these results, it was supposed that the reduction of the applied medium volume due to its exposure to CAPP might increase the concentration of RONS [34] and induce a biological impact on the analyzed cell lines (Groups I, II, V, and VI). Additionally, by increasing the organic content in the cell culture medium through the use of the Opti-MEM CAPP-activated medium, its toxic effect towards both human breast cancer cell lines was noticed. Accordingly, the exposure of a proper medium to the CAPP source for 180 s resulted in producing the CAPP-activated medium recognized as the one with a minimal toxic dose.

The anti-proliferative properties of the CAPP-activated media are currently also the focus of other research groups, and conclusions similar to our results were found in references [10,15,18,19,20]. Bekeschus et al. [10] cultured the MDA-MB-231 and SW 480 cancer cell lines in the DMEM (supplemented with 10% FBS) and directly them exposed to CAPP, which significantly reduced the viability of those cancer cells [10]. Xiang et al. showed that the media irradiated by CAPP (DMEM and DMEM/F-12) exhibited a selective character towards the highly metastatic human breast cancer cell lines [20]. Moreover, it was established that the CAPP-activated media slightly increase the cell viability of the non-cancerous MCF10A and non-metastatic MCF7 cell lines [20]. Results opposite to those reported in [20] were described by Mokhtari et al. [15]. In this case, the biological activity of the CAPP-activated DMEM was demonstrated towards different human cancer cell lines, including the human breast cancer cell lines MCF7 and SKBR3, the human lung adenocarcinoma cell line A-549, the human colon carcinoma cell line SW742, the human pancreatic cancer cell line ASPC-1, and the human primary osteogenic sarcoma cell line G-292. The biological models of the non-cancerous cell lines were also prepared using the human gland cells MCF10A and the skin fibroblast cells FMGB-1 [15]. It was concluded that the obtained CAPP-treated medium showed a significant reduction in the MCF7 cell lines cell viability, while the normal MCF10A cell lines remained untouched by CAPP for a shorter irradiation time [15]. Comparable results for these cell lines (MCF7 and MCF10A) were also presented in the research on the reduction of the cell viability following the direct CAPP treatment. In more detail, the treatment time of 60 s led to a significant decrease in the proliferation rate of the MCF7 cell line, while the MCF10A cells remained uninjured [18].

Because the FBS acts as an OH^•^ and H_2_O_2_ scavenger [35], another set of experiments was performed, where the FBS was immediately added to the proper CAPP-activated medium. The proliferation activity of the analyzed cancer cell lines (MDA-MB-231 and MCF7), incubated with the CAPP-activated medium (CAPP treatment times of 150 s, 180 s, 210 s, or 240 s), to which the FBS was added after the CAPP treatment (Groups III, IV, VII, VIII), is given in Figure 2. In the graph “**c**” (Figure 2), a comparison for the MDA-MB-231 cell line incubated in the CAPP-activated DMEM (3.0 mL, without the 3% FBS) is shown. A significant decrease in cell viability for the treatment times of 180 s and 240 s (day 1) (** *p* < 0.0014) and for the treatment times of 180 s, 210 s, and 240 s (day 2) was observed (* *p* < 0.013; * *p* < 0.013; ** *p* < 0.0014). A comparable observation was made for the CAPP-activated DMEM (1.5 mL, without the 3% FBS), as is shown in Figure 2d. Moreover, the most prominent decrease in the proliferation rate was for the 180 s and 240 s CAPP-activation times (* *p* < 0.013; ** *p* < 0.0014, respectively) of the analyzed medium. The presence of the FBS in the DMEM during the CAPP activation did not affect the cell viability of the MDA-MB-231 cell line. As can be seen in Figure 2e,f, the CAPP-activated Opti-MEM (3.0 mL or 1.5 mL, with the absence of 3% FBS) significantly affected the MDA-MB-231 cell line viability in the one-day incubation for the CAPP-activation times of 180 s and 240 s (** *p* < 0.0014; *** *p* < 0.0002). For the two-day incubation of the MDA-MB-231 cell line with the CAPP-activated Opti-MEM (3.0 mL or 1.5 mL), the respective cell viability was reduced for the medium for all the CAPP-activation times: 150 s, 180 s, 210 s, and 240 s (* *p* < 0.013; ** *p* < 0.0014; *** *p* < 0.0002). Finally, the use of the CAPP-activated DMEM (see Figure 2g,h) resulted in no difference in the case of the reduction in the cell viability of the MCF7 cell line. However, the preparation of the CAPP-activated Opti-MEM (3.0 mL) led to a decrease in the proliferation rate when the 3% FBS was not added during the CAPP-activated Opti-MEM preparation. 

Concerning the role of the addition of FBS to the CAPP-activated medium, it was established that the produced reaction mixtures significantly enhanced the toxic effect against the MDA-MB-231 as well as MCF7 cell lines. In this case, the most prominent decrease in the proliferation rate of the metastatic cancer cell line MDA-MB-231 was observed. Comparable results were described by Rodder et al. [35], who found that an increase of FBS concentration in the analyzed solutions during the CAPP treatment led to a decrease in H_2_O_2_ concentration, minimizing the death cell population [35].

#### 2.1.2. Study of the Inhibition Effect of Two Different CAPP-Activated Media on the Migration Ability 

The estimation of cell migration after the application of a proper CAPP-activated medium was assessed using the scratch test. For this test, 1.5 mL of the DMEM or the Opti-MEM, activated by CAPP for 180 s or 210 s, were selected. The times of 180 s and 210 s were chosen because they provided a minimum toxic dose. After the CAPP-activated medium production, the FBS was added to reach its final concentration of 3%. The images of the prepared scratches were acquired within 30 h of cell line treatment by the proper CAPP-activated medium. 

**Figure 3:**
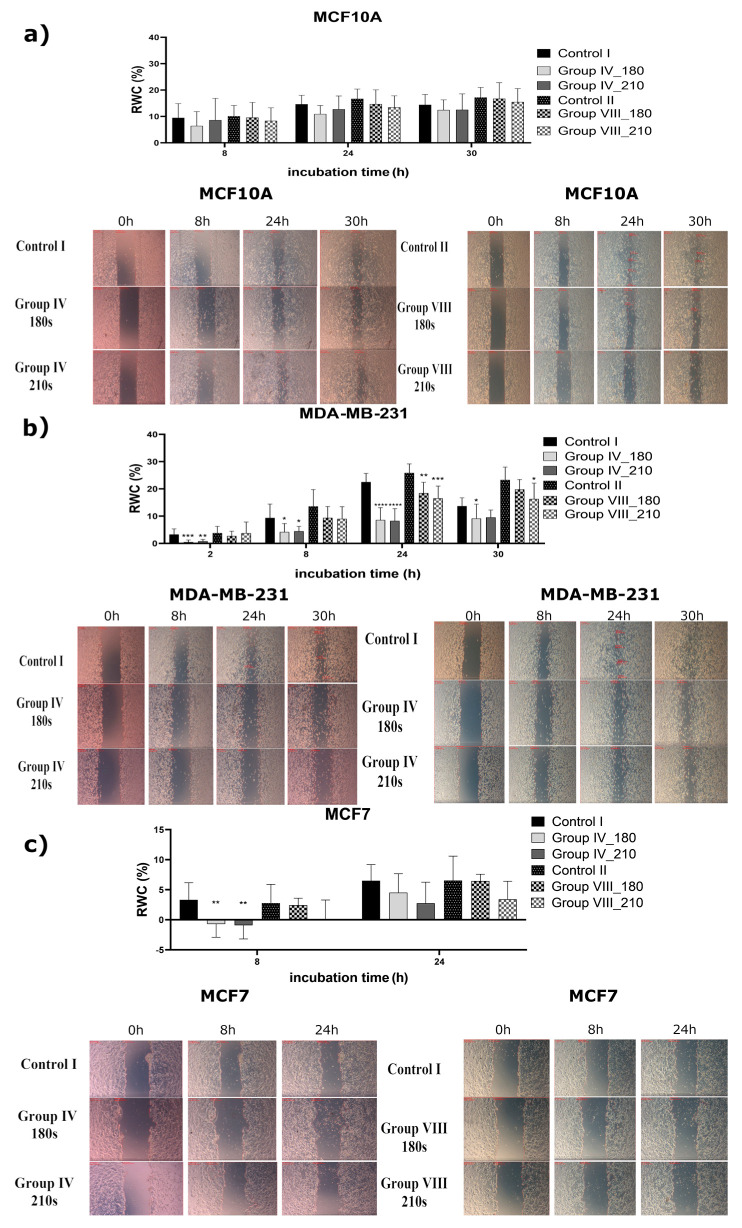
The results of the scratch test assay employed to assess the cells migration for the human breast cell line MCF10A (**a**) and the human cancer cell lines, i.e., MDA-MB-231 (**b**) and MCF7 (**c**). The grown confluent layers were scratched, and old culture media were substituted by: (i) 1.5 mL of the DMEM activated by CAPP for 180 s or 210 s (Group IV) or (ii) 1.5 mL of the Opti-MEM activated by CAPP for 180 s or 210 s (Group VIII). The relative wound closure (RWC) area was calculated following 30 h of the experiment as described in the “Materials and Methods” section. The calculated results are presented as means ± SD values for three independent experiments performed in duplicates. The statistical calculation was performed by the comparison of all the investigated groups versus the control using one-way ANOVA with the Dunnett’s post hoc test (* p < 0.016, ** p < 0.002, *** p < 0.001, **** p < 0.0001).

As can be seen from Figure 3, the scratch closure calculations of the normal human breast cell line (MCF10A) showed that the incubation in both types of CAPP-activated medium had no significant impact on cell migration, which was in line with previously presented results for the proliferation assay (see Section 2.1.1 for more details). In the case of the non-metastatic breast cancer cell line (MCF7), some significant decreases in the scratch closure area were observed for the CAPP-activated medium DMEM, following 8 h of experiments, for both selected CAPP treatment times (180 s or 210 s in case of DMEM, Figure 3c) (** *p* < 0.002, ** *p* < 0.002, respectively). For a longer treatment time (210 s) of the DMEM, a similar inhibition of the MCF7 cell line motility was found (Figure 3) (** *p* < 0.002). The experiments were conducted for 24 h, because after these CAPP treatment times, some insignificant changes in the relative wound closure area (RWC) were detected in two kinds of prepared CAPP-activated media (Figure 3). Concerning the MDA-MB-231 cell line, the most prominent reduction in cell motility was observed when the CAPP-activated DMEM was used for a shorter CAPP-activation time. In this case, the DMEM was activated by CAPP for 180 s or 210 s, and the scratch closure area was observed for 30 h (after 24 h, strong inhibition of the relative scratch closure area was detected, see Figure 3b) (**** *p* < 0.0001). Similar results were established for the Opti-MEM activated by CAPP for 180 s or 210 s (Figure 3b) (** *p* < 0.002; *** *p* < 0.001). Intriguingly, for the DMEM activated by CAPP for 180 s, the cell migration was inhibited after 30 h (* *p* < 0.016), while for the Opti-MEM activated via CAPP for 210 s; the cell migration was significantly inhibited (* *p* < 0.016). Similar results were reported by Xiang et al. [20], who studied the effect of the CAPP-activated medium on the migration ability of the MDA-MB-231 cells. Our results were also in good correlation with those reported by other research groups [22,36].

#### 2.1.3. Induction of the Programmed Cell Death

**Figure 4:**
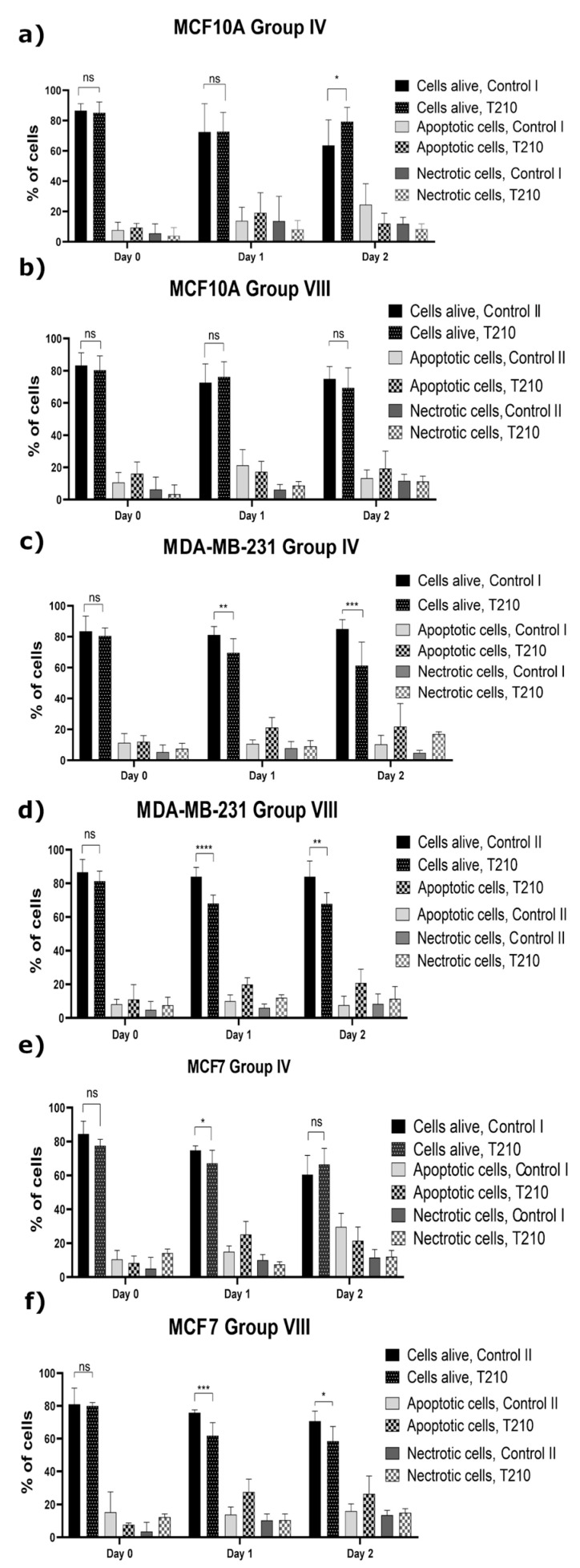
The results on the population of the alive, apoptotic, and necrotic cells of the human breast cell line MCF10A (**a**,**b**) and the human cancer cell lines, i.e., MDA-MB-231 (**c**,**d**), and MCF7 (**e**,**f**). The MCF10A (**a**), MDA-MB-231 (**c**), and MCF7 (**e**) cell lines cultured for 2 days in 1.5 mL of the CAPP-activated DMEM for 210 s (Group IV), as well as 1.5 mL of the CAPP-activated Opti-MEM for 210 s (Group VIII): MCF10A (**b**), MDA-MB-231 (**d**), MCF7 (**f**). The calculated results presented as means ± SD values for three independent experiments performed in duplicates. The statistical calculation was performed by comparison of all investigated groups versus the control using the one-way ANOVA with the Dunnett’s post hoc test (* *p* < 0.015, ** *p* < 0.0015, *** *p* < 0.0004, **** *p* < 0.0001, n.s. not significant).

In order to assess the cell death type after the application of the CAPP-activated proper medium, the Annexin V/PI test was used for the identification of the population of the apoptotic and necrotic cells (Figure 4).

The population of the alive MCF10A cells remained unchanged after culturing them in the CAPP-activated medium (Figure 4b). Additionally, the population of the alive cells incubated in the CAPP-activated DMEM for two days significantly increased as compared to the control group (CAPP treatment time of 210 s, Figure 4a) (* *p* < 0.015). Based on a culturing guide [37] for the MCF10A cell line, the DMEM culture medium is recommended, suggesting a positive effect on the normal cells. In the case of the non-metastatic breast cancer cell line (MCF7), the cells remained significantly changed in the population of the alive cells incubated in the CAPP-activated medium with a lower organic contribution in the first day of the analysis (DMEM, Figure 4e) (* *p* < 0.015). On the other hand, the application of the CAPP-activated Opti-MEM (Figure 4f) significantly decreased the percentage of the alive cells as a result of their apoptosis after one and two days of the above-described experiment (day 1—from 76.75% to 61.83%, *** *p* < 0.0004; day 2—from 74.00% to 58.50%, * *p* < 0.015). Considering the metastatic breast cancer cell line (MDA-MB-231), the presence of a lower organic matter in the DMEM (Figure 4c) resulted in a prominent induction of the apoptosis, especially after one day and two days of the culturing in the CAPP-activated medium (day 1—from 81.17% to 69.75%, ** *p* < 0.0015; day 2—from 85.00% to 60.88%, *** *p* < 0.0004). The metastatic breast cancer cell line (MDA-MB0231) treated with the CAPP-activated Opti-MEM (Figure 4d) exhibited the most prominent reduction of the alive cell population after the one-day experiment (day 1—from 84.00% to 68.12%, **** *p* < 0.0001; day 2—from 84.00% to 67.86%, ** *p* < 0.0015). It is worth mentioning that a greater reduction of the alive cells was observed in all cases for the MDA-MB-231 cell line; which was incubated in the CAPP-activated medium, following the one- and two-day experiment (** *p* < 0.002, *** *p* < 0.001, **** *p* < 0.0001, ** *p* < 0.002 respectively). The obtained results are in line with the previously presented results related to migration ability measurement, providing a good explanation of the observed phenomena (see Section 2.1.2). 

The selective induction of the apoptosis was also confirmed by others, who described the application of the CAPP-activated DMEM towards the human metastatic breast cancer MDA-MB-231 cell line [20]. Accordingly, a significant drop of the apoptosis rate was only observed in the case of the metastatic MDA-MB-231 cell line, while the MCF10A and MCF7 cells showed no differences in their apoptosis rate. For the direct CAPP treatment, it was confirmed that the irradiation induced the apoptosis in the MCF7 cell line [13,17], not damaging the MCF10A cell line [18].

**Figure 5:**
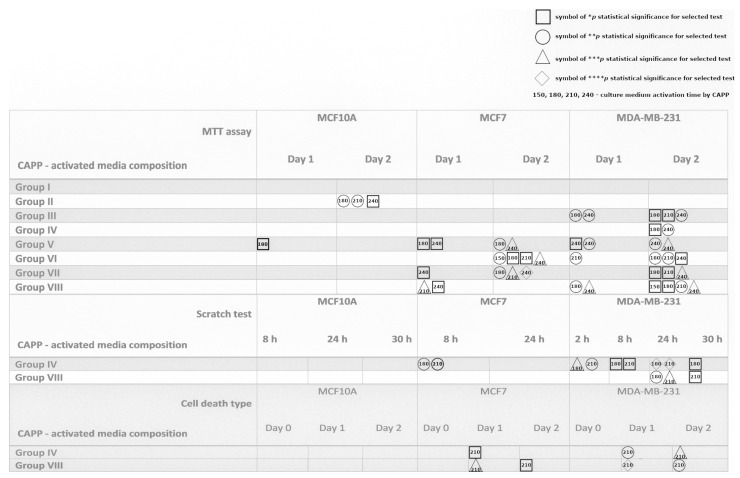
A graphical summary of the biological results successively obtained from the MTT assay (Figure 1 and Figure 2), the scratch test (Figure 3), and the cell death type appearance (Figure 4). □—the * *p* statistical significance for the selected test; ◯—the ** *p* statistical significance for the selected test; ∆—the *** *p* statistical significance for the selected test; ◊—the **** *p* statistical significance for the selected test in all the investigated CAPP-activated media compositions. The numbers given inside the symbols (150, 180, 210, and 240 s) describe the CAPP-activation times of the culture media.

Figure 5 shows a summary of the results obtained during all the biological experiments. As can be seen from the figure, in a great majority of cases, the biological activity of the MCF10A cell lines was not disturbed, showing that the CAPP-activated media has no harmful effects on the human normal cell line. Concerning the next cell line, i.e., MCF7, it can be clearly seen that any perturbations in their viability occur only for the CAPP-activated Opti-MEM, with a minimal toxic dose established to be 180 s of CAPP activation. Moreover, the generation of the CAPP-activated media in a smaller medium volume greatly impacts the activity. Additionally, the absence of the FBS during CAPP activation also improves the biological activity of the culture media. In general, interference in the cell viability increases with the time of the experiment. These observations were confirmed by the scratch test, where a significant inhibition in the cell viability was found only at the beginning of the observation and, what is quite interesting, only in the case of the CAPP-activated DMEM. However, this pattern was observed in the assessment of the cell death type, where a more significant decrease in the population of the alive cells was noted, following the first day of the experiment. The influence of the CAPP-activated media composition on MDA-MB-231 viability was as follows: The DMEM without FBS contribution during CAPP activation harms the cells with a minimal toxic dose of 180 s, indicating a major impact on the second day of the experiment. The CAPP-activated Opti-MEM leads to the disruption of cell viability in most studied groups, especially during the second day of the test, with the same minimal toxic dose. Interestingly, the cell motility was strongly inhibited throughout the experiment for the DMEM CAPP activation, while in the case of the Opti-MEM, it occurs only for a longer observation time. Finally, in the case of a decrease in the alive cell population, the results are comparable.

### 2.2. Processes and Reactions Leading to the Production of the CAPP-Activated Media with Different Biological Activities

Interactions between CAPP and liquids result in cascading reactions, leading to the production of a cocktail of various RONS. A special attention should be paid to the long-lived RONS such as NO_2_^−^, NH_4_^+^, NO_3_^−^, and H_2_O_2_. The qualitative and quantitative determination of these RONS is necessary to reveal the CAPP reactions and processes, responsible for the anticancer activity of the cell culture media activated by CAPP.

#### 2.2.1. Identification of the RONS in the Gaseous Phase of CAPP during the Production of the CAPP-Activated Media

**Figure 6:**
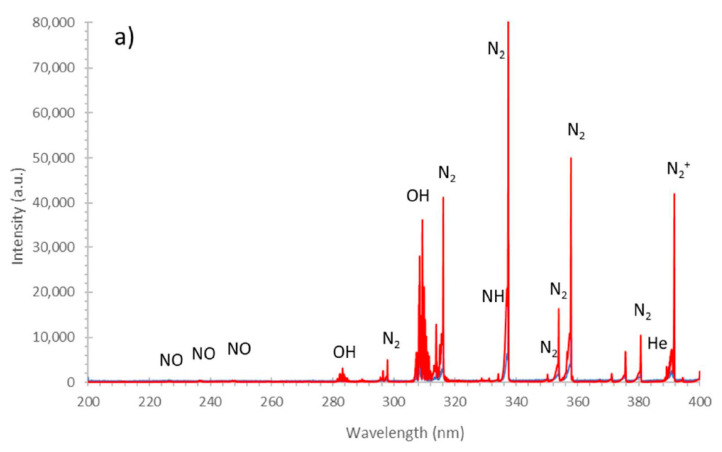

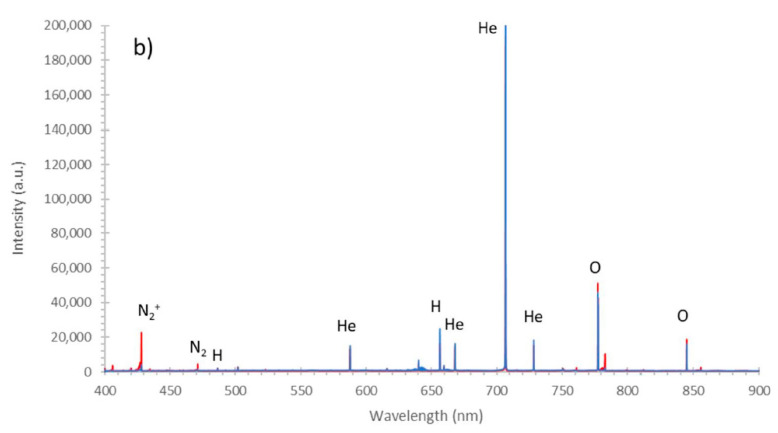
The emission spectra of CAPP generated in contact with the DMEM (red line) and the Opti-MEM (blue line) in the (**a**) 200–400 nm and (**b**) 400–900 nm spectral regions.

To identify the RONS produced in the gaseous phase of CAPP, generated during DMEM and Opti-MEM activation, optical emission spectrometry (OES) was used. As can been seen from Figure 6, quite similar RONS were identified in the case of CAPP use for the activation of both media. Accordingly, the following reactive species, i.e., NO, N_2_, N_2_^+^, NH, O, H, He, and OH, were observed in the OES spectra of CAPP (Figure 6). In the 200–260 nm region the γ-system of NO (A^2^Σ^+^−X^2^Π), with band heads at 226.9 nm (0−0), 237.0 nm (0−1), and 247.9 nm (0−3), was identified. Numerous rotational–vibrational bands of the N_2_ molecule, belonging to the C^3^Π_u_−B^3^Π_g_ system, with the most intense band heads at 315.9 nm (1−0), 337.1 nm (0−0), 357.7 nm (0−1), and 380.4 nm (0−2), were also clearly observed. The bands of the OH radical, belonging to the A^2^Σ−X^2^Π system, with the intense band heads at 309,4 nm (0−0) and 286.1 nm (0−1), were also identified. Finally, the bands of the N_2_^+^ molecule, belonging to the B^2^Σ^+^_u_ − X^2^Σ^+^_g_ system, were identified with the band heads at 391.4 nm (0−0) and 427.8 nm (0−1). Additionally, numerous lines of He I at 388.8 nm, 587.5 nm, 667.8 nm, 706.5 nm, and 728.1 nm were noted. There were also found, in the emission spectra of CAPP, during the media activation, H I lines at 486.1 nm and 656.2 nm as well as O I lines at 777.2 nm, 777.4 nm, and 844.6 nm. Based on this OES qualitative characterization, production of the selected RONS during DMEM and Opti-MEM activation was confirmed.

#### 2.2.2. Determination of the RONS Concentration in the Liquid Phase of the CAPP-Activated Medium

**Figure 7:**
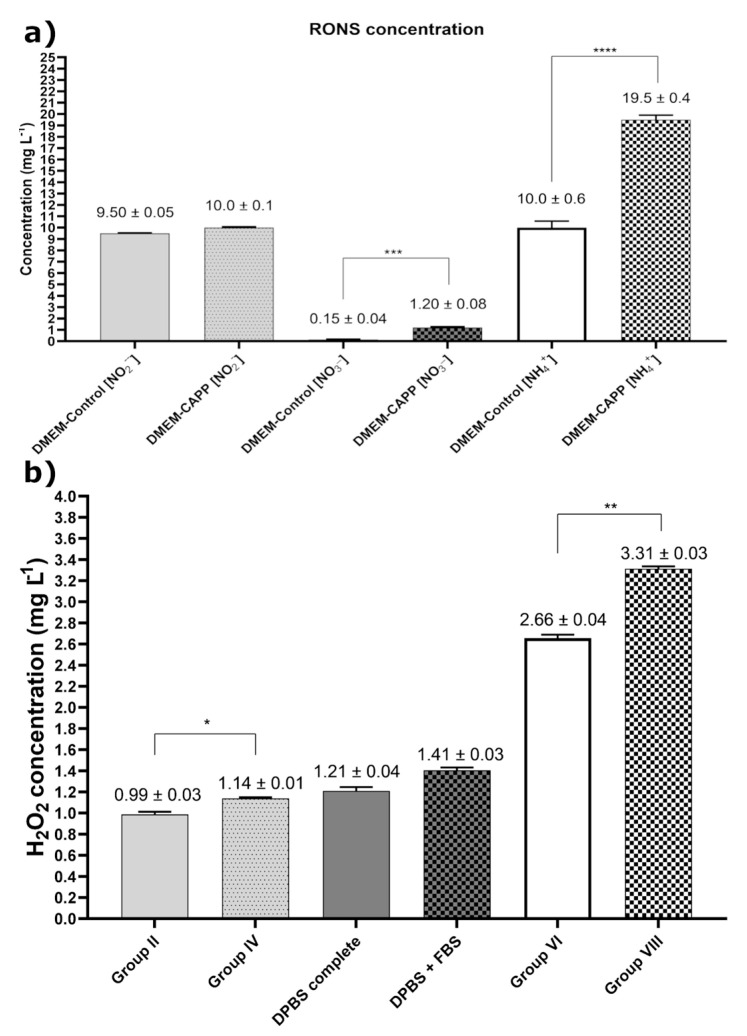
The concentrations of (**a**) the NO_2_^−^, NO_3_^−^, and NH_4_^+^ ions and (**b**) H_2_O_2_ determined in 1.50 mL of the CAPP-treated DMEM (for 180 s). The analyzed experimental groups were as follows: DMEM Groups. II (with FBS during the activation) and IV (without FBS during the activation); DPBS (with FBS during the activation), DPBS + FBS (without FBS during the activation), Groups VI (with FBS during the activation) and VIII (without FBS during the activation). The calculated results are presented as means ± SD values for three independent experiments. The statistical comparison was carried out using the one-way ANOVA analysis with the Tukey’s post hoc test (* *p* < 0.026, ** *p* < 0.009, *** *p* < 0.006, **** *p* < 0.0001).

To quantitatively determine the concentration of the selected long-term RONS, including the NO_2_^−^, NH_4_^+^, and NO_3_^−^ ions (Figure 7a) as well as H_2_O_2_ (Figure 7b), in the analyzed cell culture media, colorimetric methods were used. It was established that the concentration of all measured RONS changed after CAPP activation of the DMEM (without phenyl red). Moreover, the concentration of H_2_O_2_ varied in different CAPP-activated media. A minor change was observed for the NO_2_^−^ ions. In this case, their concentration for the CAPP-activated medium (time of the medium activation: 180 s) and the untreated one was comparable, i.e., 10.0 ± 0.1 mg L^−1^ and 9.50 ± 0.05 mg L^−1^, respectively. A much bigger change was noted for the NH_4_^+^ ions. In this case, the obtained results were as follows: 19.5 ± 0.4 mg L^−1^ and 10.0 ± 0.6 mg L^−1^ for the CAPP-activated medium (time of the medium activation: 180 s) and the untreated one, respectively (**** *p* < 0.0001). A major change was noted for the NO_3_^−^ ions. Here, the concentrations of these ions for the CAPP-activated medium (time of the medium activation: 180 s) and the untreated one were estimated to be 1.20 ± 0.08 mg L^−1^ and 0.15 ± 0.04 mg L^−1^, respectively (*** *p* < 0.006). This eight-times-higher concentration of the NO_3_^−^ ions might be partly responsible for the anticancer activity of the produced CAPP-activated medium. The CAPP activation of the culture media without the addition of the FBS following the preparation results in increased H_2_O_2_ production, while the presence of FBS during the CAPP treatment decreases the H_2_O_2_ content in all the analyzed media (1.14 ± 0.01 mg L^−1^ versus 0.99 ± 0.03 mg L^−1^ in the DMEM (* *p* < 0.026), 1.41 ± 0.10 mg L^−1^ versus 1.21 ± 0.05 mg L^−1^ in the DPBS, and 3.31 ± 0.03 mg L^−1^ versus 2.66 ± 0.04 mg L^−1^ in the Opti-MEM (** *p* < 0.009), respectively). Comparing the CAPP activation of the DMEM (Groups II and IV) to the DBPS with the lowest organic content, it appears that a higher H_2_O_2_ concentration is obtained when the organic content decreases (1.14 ± 0.01 mg L^−1^ versus 1.41 ± 0.03 mg L^−1^). However, this regularity was not observed in the case of the Opti-MEM culture media with the highest organic content (1.41 ± 0.03 mg L^−1^ versus 3.31 ± 0.03 mg L^−1^). A possible explanation for this abnormality can be associated with the color of this media, which can interfere at a maximum absorbance at 450 nm. On the other hand, the addition of the FBS to the Opti-MEM after CAPP activation significantly elevated the H_2_O_2_ concentration (2.66 ± 0.04 mg L^−1^ versus 3.31 ± 0.03 mg L^−1^; ** *p* < 0.009). The quantitative analysis of the RONS during the CAPP activation of 3.0 mL of the DMEM for 5 min was also reported by Trizio et al. [38]. Despite evident differences in the media volume and the exposition time of the DMEM to DBD, comparable to our results, differences in the concentration of the NO_2_^−^ ions (0.40 mg L^−1^ versus 0.50 mg L^−1^) and the NO_3_^−^ ions (1.90 mg L^−1^ versus 1.05 mg L^−1^) were established in the cited work in the case of the CAPP-activated medium and the untreated one. As here, the concentration of the NO_2_^−^ ions were at the same level, while in the case of the NO_3_^−^ ions, their concentration in the CAPP-activated medium was higher than that determined in the untreated medium. In addition, the concentration of the sum of the NO_3_^−^ and NO_2_^−^ ions, produced in 2.0 mL of the CAPP-activated DMEM for 120 s and used concerning the MCF7 cell line [12], was 0.52 mM, which is quite similar to our findings. Familiar concentrations of H_2_O_2_ following the CAPP treatment of the RPMI with the addition of the FBS were reported by Rodder et al. [34]. In this case, 1 mL of the RPMI with the added FBS was treated by CAPP for 20 s, which results in the production of 1.7 mg L^−1^ of H_2_O_2_. Moreover, in the paper by Yadav et al. [35], 300 s treatment of 1 mL of RPMI or MEM with the added FBS results in 1.43 mg L^−1^ and 1.56 mg L^−1^ of H_2_O_2,_ respectively. These results correlate well with our studies, where higher volumes of the culture media were irradiated by a shorter treatment time.

To reveal the impact of the media activation with the aid of CAPP on the cancer cell lines, the plasma–liquid interactions were discussed. At the beginning of the CAPP irradiation of the liquid medium, the broad spectrum of RONS was likely produced in the gas phase, including NO, N_2_, N_2_^+^, NH, O, and OH (Figure 6, according to the results collected by us) and superoxide anions (O_2_^−^), atomic oxygen (O), singlet oxygen (^1^O_2_), ozone (O_3_), oxonium ions (H_2_O^+^), in addition to the UV radiation (according to the literature reports [39,40]). These high energy molecules and reactive species interacted with different constituents in the solution, generating a broad spectrum of short life-time (peroxynitrite) and a long life-time species (H_2_O_2_, NO_2_^−^, NO_3_^−^, and organic peroxides). Because the study conducted by us was based on the indirect CAPP treatment and the preparation of the CAPP-activated media, which were then transferred into cells, the impact of the short life-time reactive species did not matter in the case of the CAPP–cells interactions. The latter reactive species likely took part in further cascade reactions, leading to an increase in the long life-time reactive species production. For this reason, in most of the published papers on the CAPP anticancer treatment, the major RONS such as NO_2_^−^, NO_3_^−^, H_2_O_2_, and organic peroxides were measured [41] as presented in Figure 7. 

Considering the different organic content of CAPP-activated culture media, it seemed that the obtained biological response and the concentration of the generated RONS could be tailored. In general, the increase of the organic contribution to a certain level resulted in elevating the production rate of H_2_O_2_, NO_2_^−^, NO_3_^−^ in various proportions [42]. On the other hand, the organic admixtures exceeding a certain level were responsible for the scavenger phenomena of certain RONS as well as an inhibited biological response of certain cell lines. A familiar situation was observed in the present study for the measurement of the H_2_O_2_ concentration (Figure 7b), where for the CAPP-activated DMEM the lowest H_2_O_2_ content was observed. In detail, for the CAPP-activated DPBS (with the lowest organic content) and the Opti-MEM, the H_2_O_2_ concentration was significantly elevated. From this perspective, the addition of the FBS during the CAPP-activation step should be considered as one of the most important factors responsible for the increased H_2_O_2_ production. Finally, the overall broad spectrum of the reactive constituents and a variability of possible by-products obtained during the CAPP activation of the complex culture medium are hard to examine in a chemical way. Therefore, the effect of the generated RONS on cells should be primarily investigated with reference to their biological response.

The presented results clearly showed that the CAPP-activated DMEM and Opti-MEM do not harm the human normal cell line while significantly they affect the cell viability of the cancer cells. This selective CAPP action can be associated with a differentiated content of cholesterol fractions in the cell membrane, playing a crucial role in maintaining membrane integrity and fluidity. Cholesterol fractions are the first barrier against the RONS, which can oxidise them, creating some holes in them for further penetration inside the cells. It was previously shown that the MCF10A cell lines, which pose a significantly lower level of cholesterol than MCF7 and MDA-MB-231 cell lines, were resistant to the MBCD-induced apoptosis, while the breast cancer cell lines were sensitive [43]. The facilitated permeability of the RONS inside the cells due to membrane oxidation what creates some holes, results in the accumulation of, e.g., H_2_O_2_, leading to oxidative stress, which is greatly harmful for the MCF7 and MDA-MB-231 cell lines but not for the MCF10A cell line [44]. Additionally, cancer cells abundantly express aquaporins (AQP) as compared to normal cells, i.e., AQP1, AQP3, and AQP5. Aquaporins, being membrane proteins that create channels in the membrane, facilitate the transport of water and glycerol, and correlate with the metastatic character of human breast cancer cells and their aggressiveness [45]. They can also promote H_2_O_2_ permeation [46]. Finally, it was confirmed that the targeted therapy related to a mutant p53 gene is a promising treatment against breast cancer and can be correlated with observed CAPP-activated DMEM and Opti-MEM phenomena [47].

## 3. Materials and Methods 

### 3.1. CAPP-Based Reaction-Discharge System Used for CAPP-Activated Media Production

**Figure 8:**
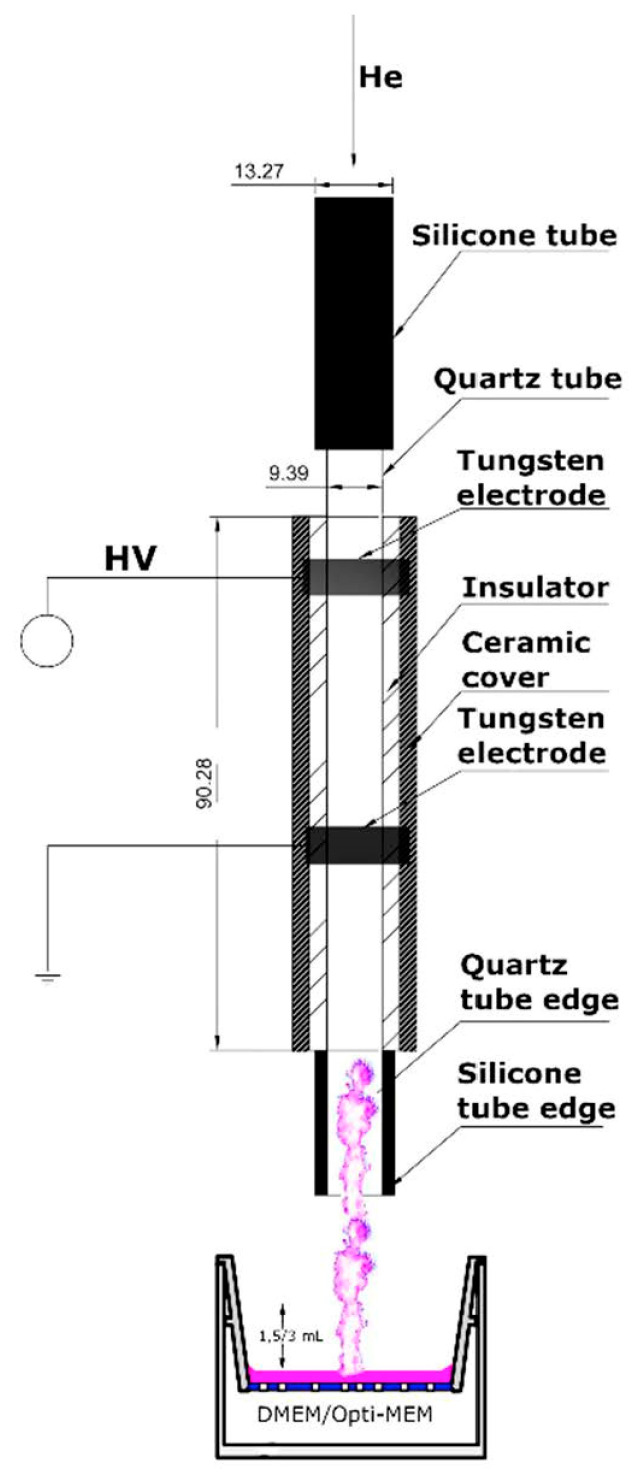
The CAPP-based reaction-discharge system used for the CAPP-activated media production.

To obtain the CAPP-activated media, including the DMEM and the Opti-MEM, a CAPP-based reaction-discharge system, previously developed and optimized in our research group, was used [33]. In this CAPP-based reaction-discharge system, a DBD plasma jet is sustained in He and used as a CAPP source. As is shown in Figure 8, the main corpus of the applied CAPP-based reaction-discharge system consisted of an E-57 epoxy resin with an immersed quartz tube and two ring-shaped tungsten electrodes attached. From the inside, the corpus was covered with a CORIAN insulator packed into a ceramic material. The He-CAPP cone was formed and extended beyond the ceramic cover as much as 38 mm. A HV potential was supplied to the reaction-discharge system using a portable power supply (Dora Electronics Equipment, Wilczyce, Poland). The optimal operating conditions of this reaction-discharge system, leading to the CAPP-activated media production, were as follows: The frequency of the modulation: 2.14 kHz; the duty cycle: 74.29%; and the He flow rate 10.6 L min^−1^. Under these optimal operating conditions, the gas temperature (based on the OH (0-0) radical emission spectrum) was about 37 °C (310 K) [33]. The additional electrical parameters, i.e., the voltage amplitude, the voltage waveform shape, and the general frequency, were assessed using a digital two-channel storage oscilloscope (Tektronix, TBS 1000, Beaverton, OR, USA) and were as follows: (i) the voltage amplitude: 6 kV; (ii) the voltage waveform: square wave; and (iii) the general frequency: 66.45 kHz. The distance between the activated medium and the tip of CAPP was measured using a digital caliper and was equal to 25.00 mm. 

### 3.2. Breast Cancer Cell Lines and Their Culture Conditions

To assess the biological effects of the CAPP-activated media in the in vitro models of breast cancer, two cell lines were selected: The human non-metastatic breast adenocarcinoma MCF7 (ATCC^®^ HTB-22TM) cell line [48] and the human metastatic MDA-MB-231 (ATCC^®^ HTB-22TM) cell line (delivered from a pleural effusion of a middle-aged Caucasian female) [49]. The MCF 7 cell line is non-invasive and exhibits a low proliferation rate in contrast to the MDA-MB-231 cell line, which is considered highly aggressive and invasive, possessing elevated expression of p53 protein. Furthermore, the non-cancerous human normal MCF10A cell line (ATCC^®^ CRL-10317TM), originating from the spontaneously immortalized benign proliferative breast tissue, was chosen as the non-carcinogenic in vitro model [50]. All the analyzed cell lines were cultured in the Opti-MEM with the GlutaMAX medium (Thermo Fisher Scientific Inc., Grand Island, NE; New York, NY, USA), supplemented with 3% of the FBS (Gibco, Origin: Brazil, Campinas, Brazil), a 100 U mL^−1^ penicillin solution (Sigma-Aldrich, Steinheim, Germany), and a 100 μg mL^−1^ streptomycin solution (Sigma-Aldrich, Steinheim, Germany). Additionally, the normal MCF10A cell line was supplemented with insulin (Sigma-Aldrich, Steinheim, Germany), an epidermal growth factor (EGF, 20 μg mL^−1^), a CorningTM Endothelial Cell Growth Supplement (ECGS, 50 μg mL^−1^), and hydrocortisone (0.5 μg mL^−1^). EGF, ECGS, and hydrocortisone were obtained from Sigma-Aldrich (Sigma-Aldrich, Steinheim, Germany). The analyzed cell lines were incubated under the following conditions: Temperature 37 °C, 5% CO_2_, and 95% air atmosphere. After the preparation and incubation of the cell lines, they were routinely passaged using a 0.05% Trypsin/0.02% EDTA (*w*/*v*) solution (IITE PAN, Wroclaw, Poland). 

To evaluate the effect of the selected CAPP-activated media (see Table 1 for more details) on the biological activity of cells, including their viability, migration rate, and the type of cell death, the following procedure was used. Firstly, the defined volume (3.0 mL or 1.5 mL) of the selected media (DMEM or Opti-MEM with GlutaMAX), supplemented with a 100 U mL^−1^ penicillin solution, and a 100 μg mL^−1^ streptomycin solution, were introduced to the 12-well plates. Considering the hypothesis that the presence of the FBS in the culture medium during the CAPP treatment can act as a scavenger of H_2_O_2_, in the first step of the experiments (Table 1, experimental Groups I, II, V, and VI), 3% of the FBS was added to the medium before the CAPP treatment, opposite to the second step of the experiments in which 3% of the FBS was introduced to the analyzed media immediately after the CAPP treatment (Table 1, experimental Groups III, IV, VII, and VIII).

In order to produce the particular type of CAPP-activated medium (see Table 1), the CAPP-based reaction-discharge system was used (see Section 3.1, CAPP-based reaction-discharge system used for CAPP-activated media production, for more details). The distance between the tip of the CAPP and the analyzed medium was set to 25.0 mm, remaining in direct contact with the culture media. The medium was activated by CAPP within 45 s, 90 s, 120 s, 150 s, 180 s, 210 s, or 240 s. Finally, the freshly prepared CAPP-activated medium (supplemented before or after the CAPP activation with 3% of the FBS) was collected in separate plastic tubes and then directly transferred into the prepared cell lines for the further biological analysis.

### 3.3. Biological Activities of the CAPP-Activated Media Concerning the Human Breast Cancer Cell Lines

#### 3.3.1. Determination of the Cell Viability

In order to determinate the influence of the CAPP-activated media on cell viability, an MTT (3-(4.5-dimethylthialzol-2-yl)-2.5-diphenyl tetrazolium bromide) (Thermo Fisher Scientific Inc., Grand Island, NE, New York, NY, USA) assay was carried out [51]. Initially, two cell lines were chosen: MCF7 and MDA-MB-231 (see Section 3.2, breast cell lines and their culture conditions, for more details). As a control, the non-cancerous MCF10A cell line was chosen. The MTT assay was performed as follows: 5 × 10^3^ of given cells (per well) were plated in 100 μL (per well) of the complete medium in the flat-bottomed 96-well plates. Then, the cells were incubated at 37 °C, with 5% CO_2_ and 95% air atmosphere, for 24 h. After this time, the completed medium was carefully removed from each well and refilled with a defined volume of the proper CAPP-activated medium (Table 1, experimental groups, Groups I, II, V, and VI), or with the CAPP-activated medium supplemented with 3% of the FBS (Table 1, experimental groups, Groups III, IV, VII, and VIII) for the indicated time period (day 0, day 1, and day 2). Next, 10 μL of the MTT reagent was added to each well to reach a final concentration of 10 mg mL^−1^. Then, the obtained reaction mixtures were incubated for 3 h at 37 °C, with 5% CO_2_ and 95% air atmosphere, maintaining darkness. After 3 h of incubation, the obtained reaction mixture was thoroughly removed from each well and carefully dried. Empty wells were filled with 100 μL of DMSO (POCH SA, Gliwice, Poland) per well. After 45 s of the reaction with DMSO, a purple color appeared in each well. The described change in color of the analyzed reaction mixtures was associated with the formation of formazan crystals. Next, the absorbance of the latter mixtures at 570 nm was measured using a Victor 2 microplate reader (Perkin Elmer, Woodbridge, Vaughan, ON, Canada). The experiments were done in triplicate at least three times.

#### 3.3.2. Determination of the Cell Migration

To assess the influence of the CAPP-activated media on cell migration, a scratch test was conducted [52]. Two breast cancer cell lines were chosen: MCF7 and MDA-MB-231 (see Section 3.2, breast cell lines and their culture conditions, for more details), and the MCF10A cell line served as a control. Firstly, the cell lines, with a concentration of 1.5 × 10^5^ cells per well, were separately seeded into the 24-well plates. Next, the incubated cells were suspended in 350 μL of the Opti-MEM culture medium supplemented with 3% of the FBS, a 100 U mL^−1^ penicillin solution, and a 100 μg mL^−1^ streptomycin solution, and cultured to produce a confluent cell monolayer. Then, the cells were incubated for 24 h at 37 °C, with 5% CO_2_ and 95% air atmosphere. After this time, the confluent surfaces of the cells were scratched using a 200 µL sterile pipette tip to prepare the straight dashes for the scratch assay. Then, the cells detached during the scratch preparation were removed with the complete culture medium. Next, the attached cell monolayer was filled using 350 μL of the proper CAPP-activated media (see Table 1 for more details) treated by CAPP either for 180 s or 210 s. Afterwards, the 24-well plates, containing cell monolayers, were incubated for 30 h at 37 °C, under the 5% CO_2_ and 95% air atmosphere. The experiments were carried out three times in duplicate. The cell migration was acquired by taking digital pictures at 0, 2, 8, 24, and 30 h after the scratches. The closure area was calculated using ZEN 3.1 blue edition software (Carl Zeiss Microscopy GmbH, Jena, Germany) and presented as the relative wound closure (RWC, %), as previously described in reference [53].

#### 3.3.3. Estimation of the Cell Death Type

To assess the cytotoxic effect of the CAPP-activated media on the analyzed human breast cancer cell lines, the cell death Annexin V/PI test was performed (eBioscienceTM Annexin V Apoptosis Detection kit APC, Invitrogen, Thermo Fisher Scientific Inc., Grand Island, NE; New York, NY, USA) [54,55]. In this experiment, the proper cell lines, including MCF7, MDA-MB-231, and MCF10A, were seeded onto the 24-well plates with a density of the 1.5 × 10^5^ cells per well. Then, the prepared cell lines were suspended in 350 μL of the Opti-MEM medium supplemented with 3% of the FBS, a 100 U mL^−1^ penicillin solution, and a 100 μg mL^−1^ streptomycin solution. Next, the seeded well plates were incubated for 30 h at 37 °C, with 5% CO_2_ and 95% air atmosphere. After 30 h of incubation, the complete medium was removed from each well and refilled by the freshly CAPP-activated medium, prepared after 210 s irradiation (Table 1, experimental groups, Groups III, IV, VII, and VIII). Afterwards, all the cells from the 24-well plates were detached by using a 0.05% Trypsin/0.02% EDTA solution and washed, firstly with 1.00 mL of PBS (Gibco, Thermo Fisher Scientific Inc., Grand Island, NE; New York, NY, USA) and secondly with a buffer binding solution (included in kits, eBioscienceTM Annexin V Apoptosis Detection kit APC, Invitrogen, Thermo Fisher Scientific Inc., Grand Island, NE; New York, NY, USA). Next, the cell pellets were vigorously agitated and suspended in an Annexin V dye solution (according to the instructions) and then incubated for 15 min at room temperature. After the incubation, the resultant solutions were filled with 1.00 mL of the buffer binding solution and then centrifuged. In the final step, propidium iodide (PI, according to the instructions) was added to the analyzed pellets. The same procedure was employed in the case of the cells not treated with the CAPP-activated media (controls). However, after the previously described procedure of cell seeding and incubation, the complete medium was replaced by a fresh one. The prepared controls were further used for the Annexin V/PI assay, as was described before. Next, the stained cells were assessed by a FACS-Calibur flow cytometry instrument (Becton-Dickinson, Franklin Lakes, NJ, USA). The apoptotic cell population (Annexin V positive, PI negative), the necrotic cells (Annexin V negative, PI positive), and the alive cells (Annexin V negative, PI negative) were measured using the FL4 (λ_em_ = 660 nm) and FL2 (λ_em_ = 535 nm) modes. The results were analyzed using a Flowing Software 2 program (Flowing Software ver. 2.5.1, Flowing Software, Turku, Finland). The same experimental protocol was conducted for the group of cell lines exposed to the CAPP-activated media for 24 h and 48 h. The results were presented as means ±SD values for three independent experiments, each carried out in duplicates. In order to perform the statistical analyses, one-way ANOVA was applied. Additionally, GraphPad Prism 8 software (GraphPad Prism version 8.0.1 for Windows, GraphPad Software, San Diego, CA, USA) was employed to present the final results.

### 3.4. Estimation of the CAPP-Derived Active Constituents Leading to the Anticancer Activity of the Analyzed Media

For the qualitative and quantitative determination of the RONS, the 1.50 mL portions of the analyzed media were placed into the wells of the 24-well plates, and supplemented with a 100 U mL^−1^ penicillin solution and a 100 μg mL^−1^ streptomycin solution. Next, the analyzed media were activated by CAPP, operated under the working conditions given in Section 3.1, CAPP-based reaction-discharge system used for CAPP-activated media production. During the CAPP activation, the qualitative determination of the RONS in the gas phase produced during the CAPP operation was conducted using the OES measurements. On the other hand, the quantitative measurement of these species in the liquid phase was performed after 180 s of the proper medium irradiation by CAPP.

#### 3.4.1. Identification of the Reactive Oxygen and Nitrogen Species Using Optical Emission Spectrometry

The OES measurements were made to identify the RONS generated in the plasma-gas phase as a result of the CAPP–liquid (medium) interactions and acquired during the medium activation by CAPP. The interactions between CAPP and DMEM (without 3% FBS) and between CAPP and Opti-MEM (without 3% FBS) were analyzed. The radiation emitted by the CAPP system in the near-liquid surface zone was imaged with the aid of a UV achromatic lens (f = 60) on the entrance slit (10 µm) of a Shamrock SR-500i spectrometer (Andor, Belfast, United Kingdom). An OES spectrometer was equipped with two holographic gratings (1800 and 1200 lines nm^−1^) and a Newton DU-920P-0E (Andor, Belfast, United Kingdom) CCD camera. The data processing and the OES spectrometer acquisition parameters were maintained with Solis software (Andor, Belfast, United Kingdom). The CCD camera was operated in the full vertical binning (FVB) mode; a 1-s integration time was applied. The OES spectra were acquired in the 200–900 nm spectral range. For the spectral range above 400 nm, a PG-5 filter (Zeiss Jena, Jena, Germany) was additionally applied for eliminating second order radiation.

#### 3.4.2. Determination of the Selected RONS Concentration Produced in the CAPP-Activated Media 

The colorimetric methods were used to quantitatively determine the concentration of the NO_2_^−^, NO_3_^−^, and NH_4_^+^ ions in the CAPP-treated (for 180 s) DMEM. In this case, the CAPP activation was performed for the DMEM without the phenol red dye because it is dark-pink and could affect the colorimetric measurements. For that reason, the colorless medium, which has a composition quite similar to the complete DMEM, was used. The following protocols were taken for the determination of the RONS concentration:

(i) NO_2_^−^ ions: To determinate the concentration of the NO_2_^−^ ions in the CAPP-activated medium, a HANNA HI 96708 spectrophotometer (HANNA Instruments, Olsztyn, Poland) was used. The measurements were performed according to the protocol suggested by the manufacturer and applying all the reagents and solutions provided by the manufacturer. As a control, the content of the NO_2_^−^ ions in the medium not activated by CAPP was assessed.
(ii) NO_3_^−^ ions: To assess the concentration of the NO_3_^−^ ions in the CAPP-activated medium, a HANNA HI 96728 spectrophotometer (HANNA Instruments, Olsztyn, Poland) was used. The measurements were conducted according to the protocol suggested by the manufacturer and using all the reagents and solutions provided by the manufacturer. As a control, the content of the NO_3_^−^ ions in the medium not activated by CAPP was assessed.
(iii) NH_4_^+^ ions: To estimate the content of the NH_4_^+^ ions in the analyzed medium, Nessler’s method was used [56]. In this method, the reaction with Nessler’s (K_2_HgI_4_) reagent (Sigma-Aldrich, Steinheim, Germany) was used for the determination of the NH_4_^+^ ions. In this case, the absorbance of the obtained product, i.e., [(Hg-O-Hg)NH_2_], was measured spectrophotometrically at 420 nm, using an Analytik Jena AG UV/Vis Specord 210 Plus (Jena, Germany). External calibrations with the simple standard solutions were used for the quantification. As a control, the content of the NH_4_^+^ ions in the medium not activated by CAPP was assessed. All experiments were performed in triplicates.
(iv) H_2_O_2_: To measure the total concentration of H_2_O_2_ in the prepared CAPP-activated media, the spectrophotometric method with ammonium metavanadate (NH_4_VO_3_) was adopted [57]. Following the reaction of H_2_O_2_ with NH_4_VO_3_ (Avantor Pefrormance Materials, Gliwice, Poland) in a H_2_SO_4_ solution (Avantor Pefrormance Materials, Gliwice, Poland), the absorbance of the resultant peroxovanadium cations formed in the solutions was measured at 450 nm with the aid of an Analytik Jena AG UV/Vis Specord 210 Plus (Jena, Germany). The contents of NH_4_VO_3_ and H_2_SO_4_ in these solutions were 6.2 mmol L^−1^ and 0.058 mol L^−1^, respectively. The H_2_O_2_ concentration was calculated in the culture medium with the organic content and in a DPBS solution. The external calibrations with the simple standard solutions were used for the quantification. As a control, the content of H_2_O_2_ in the respective procedural blank solution was assessed.

### 3.5. Statistical Analysis

The graphs (Figure 1, Figure 2, Figure 3 and Figure 4) and the statistical analysis for all the biological tests were performed using Prism 8.0 (GraphPad Software, San Diego, CA, USA). The comparison of the investigated groups versus the control group was made using one-way ANOVA with Dunnett’s post hoc test. Figure 7 was constructed and the relevant statistical analyses for the chemical analysis were performed using Prism 8.0 (GraphPad Software, San Diego, CA, USA) as well. The comparison of the investigated groups versus the controls group was made using one-way ANOVA with Tukey’s post hoc test. 

## 4. Conclusions

In the present study, we tried to answer the question whether the chemical composition of the CAPP-treated media is important for their anticancer activity towards the breast cancer cell lines or not. Based on the present research, it was found that the chemical composition of the CAPP-activated medium is indeed important for their anticancer activity. More specifically:

(i) The resultant CAPP-activated medium did not exhibit the apoptotic effect on the normal MCF10A cell line, developing an opportunity to successfully design a selective approach against the human breast cancer cells.
(ii) The resultant CAPP-activated medium had a harmful effect on the MCF7 and MDA-MB-231 cancer cell lines.
(iii) The presence of the FBS during the CAPP-activated media preparation negatively affected the biological response of the MCT7 and MDA-MB-231 cell lines, causing a minor decrease in their viability and disrupting the cell viability of the MCF10A cells.
(iv) The choice of the proper culture medium for the production of the CAPP-activated media with the highest biological impact is a crucial step. For the selected biological models, the culture medium with a lower content of the organic matter in the CAPP-activated DMEM resulted in a significant drop in the cell viability of the MCF7 and MDA-MB-231 cancer cells as well as in the inhibition of their motility.
(v) The disturbance in the life processes of the breast cancer cell lines was associated with the induction of the apoptosis by the CAPP-activated media. The largest population of the cells with the apoptotic pathway as well as the strongest inhibition in the cell migration was observed for the MDA-MB-231 cancer cells. This led us to the conclusion that this cell line is more sensitive to the CAPP-activated media. We believe that the studies carried out by us could be the base for alternative therapy, dedicated to the highly aggressive human breast cancer.

## 5. Patents

The construction of the portable He-DBD-based reaction-discharge system is protected by Polish Patent Application no. P 429275 (UP RP, 14 March 2019).

## Figures and Tables

**Table 1:** The summary of the CAPP-activated media composition.

Group	CAPP-Activated Media Preparation Procedure
Control I	Cell incubated in a complete untreated culture medium DMEM
Control II	Cell incubated in a complete untreated culture medium Opti-MEM
Group I	Cell incubated in the DMEM irradiated in volume 3.0 mL supplemented with 3% FBS
Group II	Cell incubated in the DMEM irradiated in volume 1.5 mL supplemented with 3% FBS
Group III	Cell incubated in the DMEM irradiated in volume 3.0 mL without the 3% FBS
Group IV	Cell incubated in the DMEM irradiated in volume 1.5 mL without the 3% FBS
Group V	Cell incubated in the Opti-MEM irradiated in volume 3.0 mL supplemented with 3% FBS
Group VI	Cell incubated in the Opti-MEM irradiated in volume 1.5 mL supplemented with 3% FBS
Group VII	Cell incubated in the Opti-MEM irradiated in volume 3.0 mL without 3% FBS
Group VIII	Cell incubated in the Opti-MEM irradiated in volume 1.5 mL without 3% FBS
